# Leveraging extreme scale analytics, AI and digital twins for maritime digitalization: the VesselAI architecture

**DOI:** 10.3389/fdata.2023.1220348

**Published:** 2023-07-27

**Authors:** Loukas Ilias, Giannis Tsapelas, Panagiotis Kapsalis, Vasilis Michalakopoulos, Giorgos Kormpakis, Spiros Mouzakitis, Dimitris Askounis

**Affiliations:** Decision Support Systems Laboratory, School of Electrical and Computer Engineering, National Technical University of Athens, Athens, Greece

**Keywords:** maritime, big data, artificial intelligence, extreme-scale analytics, distributed systems, system architecture

## Abstract

The modern maritime industry is producing data at an unprecedented rate. The capturing and processing of such data is integral to create added value for maritime companies and other maritime stakeholders, but their true potential can only be unlocked by innovative technologies such as extreme-scale analytics, AI, and digital twins, given that existing systems and traditional approaches are unable to effectively collect, store, and process big data. Such innovative systems are not only projected to effectively deal with maritime big data but to also create various tools that can assist maritime companies, in an evolving and complex environment that requires maritime vessels to increase their overall safety and performance and reduce their consumption and emissions. An integral challenge for developing these next-generation maritime applications lies in effectively combining and incorporating the aforementioned innovative technologies in an integrated system. Under this context, the current paper presents the architecture of VesselAI, an EU-funded project that aims to develop, validate, and demonstrate a novel holistic framework based on a combination of the state-of-the-art HPC, Big Data and AI technologies, capable of performing extreme-scale and distributed analytics for fuelling the next-generation digital twins in maritime applications and beyond.

## 1. Introduction

The modern maritime industry is producing data at an unprecedented rate, given that naval vessels are now equipped with an ecosystem of sensors that capture data related to the condition of a ship (performance, fuel consumption, emissions etc.) as well as external conditions (e.g., weather) (Lytra et al., 2017). The capturing and processing of such data is integral to create added value for maritime companies and other maritime stakeholders. While traditional approaches do not have the capacity to uncover the true potential of big data, innovative technologies, techniques, and approaches that were conceived for big data management are required to deal with the increasing data volumes (Pau et al., 2022). Indicative technologies that must be leveraged by maritime stakeholders to develop the next generation of maritime systems and applications are extreme-scale analytics, artificial intelligence (AI) and high-powered computing among others (Mouzakitis et al., 2023). Such innovative systems are not only projected to effectively deal with maritime big data but to also create various tools that can assist maritime industries, in an evolving and complex environment, to improve overall safety and reduce naval accidents and human casualties caused by human errors, improve ship performance, optimize naval routes and fleet intelligence, and reduce fuel consumption and Greenhouse Gas (GHG) emissions (Mouzakitis et al., 2022). The aforementioned needs require innovative solutions and smarter systems, which can be realized through AI, Machine Learning (ML), Deep Learning (DL), digital twins and other digital technologies. Capitalizing on these innovative tools in existing maritime processes requires the incorporation of various advanced technologies into an integrated system in a way that leverages both the increased capabilities of the individual technology but also and more importantly the not yet fully explored capabilities that stem from their combination. For this reason, there is a growing interest from both the research and the commercial community to create system architectures that can effectively combine big data and AI, while taking into consideration distributed computing, HPC, AI and other technologies that require large amounts of data to effectively operate and create value.

Stemming from the above, this study presents the architecture of VesselAI, an EU-funded project that aims to develop, validate and demonstrate a novel holistic framework based on a combination of the state-of-the-art HPC, Big Data and AI technologies, capable of performing extreme-scale and distributed analytics for fuelling the next-generation digital twins in maritime applications and beyond, including vessel motion and behavior modeling, analysis and prediction, ship energy system design and optimization, unmanned vessels, route optimization and fleet intelligence.

Below we mention some requirements, which have been identified and used to drive the design of the VesselAI architecture:

- Data collection and management: The VesselAI platform should be able to collect/store large volumes of historical data, ingest real-time data from different data sources like AIS, weather and geospatial data.
- Semantic enrichment and reasoning engine: The harmonization of heterogeneous datasets is a critical functionality that must be covered from VesselAI system as also to extract hidden insights and patterns through maritime data semantic representations.
- High performance computing for job scheduling and parallelization: Acceleration infrastructure, like NNP, VPU and GPU will be used to provide workflow scheduling and scheduling, as well as parallelization will enable the scaling of performance in extreme scenarios and provide inference and algorithms training instantaneously.
- AI models and analytics: VesselAI must offer a fully customized scientific environment that aids Data Scientists and Maritime Analysts to train data driven models, store them and in general to manage models lifecycle.
- Advanced visualizations and reports engine/analytics environment: Set of pipelines to monitor Machine Learning/Deep Learning pipelines, to configure data engineering and inference jobs and offer data selection, filtering, aggregation and attribute selection.
- Security and access control: The platform should provide state of the art mechanisms that enable security and access control protocols like OAuth 2.0 and UMA-2.0, to provide finer grained authentication and authorization to ICT system end users.

This paper is structured as follows: Section 1 provides the introduction to the thematic area and the scope of the study. In Section 2, indicative bibliographic research on four key technological areas is presented. Section 3 presents the VesselAI concept and architecture and finally, Section 4 concludes the document and presents future pathways for expanding on the current study.

## 2. Related work

### 2.1. Extreme scale processing architectures

Distributed and extreme-scale processing architectures are in the forefront of big data stream processing and High-Performance Computing (HPC). Stream processing is the processing of data that is ingested in real-time or near real-time (Akbar et al., 2015). The majority of research publications apply approaches in which IoT generated data are ingested directly by a cloud infrastructure despite the shortcomings of the technology when it comes to efficiently handling large data streams (Cao and Wachowicz, 2019a). To address big data dimensions such as volume and velocity, the Lambda architecture was introduced (Warren and Marz, 2015), which is a cloud architecture that deals with traditional issues of big data volumes stemming from IoT applications as it provides much better scalability and fault tolerance (Lin, 2017). On the other hand, the Kappa Architecture is centered around simplicity. For that reason, this approach replaces the batch processor component with a streaming processor that is much better suited to deal with data streams in a cloud infrastructure due to higher data rates and retention times despite the fact that it requires a larger in-memory storage space (Wingerath et al., 2016). Finally, a new IoT architecture was proposed by Cao and Wachowicz (2019b), that focuses on the optimization of analytical tasks in IoT data streams and on uncovering useful insights about the real-world (from where data are ingested in practice via the IoT sensors), both for better understanding what is happening (what do the data tell us) as well as improving the accuracy of predictions that are produced by the system. This process was named the Analytics Everywhere model and its primary focus is the automation of analytical tasks that are known in prior.

### 2.2. Distributed data storage and management

The maritime domain is a remarkably heterogeneous environment. Maritime data processing applications often must incorporate information from many different data sets, such as ship positions and movements, measurements from various maritime devices and their metadata, weather information, geospatial data, and environmental information (Lytra et al., 2017). The data can come in the form of streaming data or batch data, and they differ in volume, velocity, variety, variability, veracity (Ishwarappa and Anuradha, 2015) and formats. Another outstanding property of the maritime domain is the diversity of the devices involved. A broad range of IoT sensors produce a vast amount of streaming data, which are often first (temporarily) stored, cleansed, filtered, and aggregated on edge devices before being sent upstream to fog devices, cloud instances or bare-metal server machines for their further use (e.g., advance analysis powered by AI technologies and visualization). There are five stages in (maritime) data storage and processing workflow: data collection, data cleansing, data storage, data processing, and data emission. In the following paragraph, indicative approaches and technologies for each stage are briefly presented.

For data collection, Apache Kafka is widely used as a distributed system that can draw data from various sources, whether they contain batch or streaming data. Data cleansing in the maritime domain mainly focuses on AIS datasets, stemming from the fact that AIS is an old technology that was not designed to fit the big data paradigm (Svanberg et al., 2019). One of the main challenges in cleansing AIS is the correction of data inconsistencies, which some researchers (Abdallah et al., 2019) tried to solve by applying text analytics and natural language processing. For data storage, Distributed File Systems (DFSs) are widely used as they allow data storage in multiple remote nodes, while allowing data access as if the data was stored locally. In practice, Hadoop Distributed File Systems (HDFSs) (Shvachko et al., 2010) are one of the most popular solutions for systems that utilize big data. For data processing and emission in distributed systems, frameworks such as Apache Hive and Presto are utilized, offering querying and analytics capabilities over large quantities of distributed data.

### 2.3. Advanced ML and DL optimization techniques

Research on Machine Learning (ML) and Deep Learning (DL) models has created impressive achievements in the past few years. Modern vessel positioning sensors produce large quantities of data that can improve maritime knowledge and applications. Vessel positioning data are dynamic, continuous, and sparse on time and space, requiring methods capable of handling spatiotemporal data to derive and learn the hidden knowledge. Over the last decade, ML-based methods, and especially Artificial Neural Networks (ANNs) have attracted a renewed research interest to train large models on vast amounts of spatiotemporal-related data to solve various difficult problems, due to the advancements of Deep Learning (DL) methods and GPS-based technologies. Recent literature reviews have highlighted that several papers employ the power of the special Recurrent Neural Network (RNN) based architectures, which have become the State of the Art (SotA) for temporal modeling.

The current status of the research bibliography in DL theory and architectures, in general, is given in a comprehensive survey (Alom et al., 2019). A detailed review of the SotA in applying DL techniques for various spatiotemporal data mining tasks is presented in Wang et al. (2022), while a systematic review of ML methods for spatiotemporal sequence forecasting tasks is provided in Shi and Yeung (2018). Focusing on the maritime domain, ANNs and generally ML methods have been used to solve various complex problems. The current status of SotA navigation methodologies in the maritime intelligence research field is presented in Tu et al. (2018). However, most of these works do not take into account the entire range of special characteristics or requirements of maritime real applications, while the impact of ML methods on the maritime industry remains largely unknown.

### 2.4. Simulation modeling and AI, data-driven techniques for applications and digital twins in the maritime domain

Properly analyzing and predicting various aspects of a maritime vessel's behavior is integral in maritime research as it can provide the next generation of maritime applications with use cases that include simulations, vessel traffic management applications, route planning and optimization, among others. The concept of co-simulation is referring to the modeling and simulation of different subsystems in a distributed way, which are composed and as a whole form and more generic simulation model. The need for co-simulation arises, when large systems consist of different parts, which are modeled and simulated by different techniques tools or algorithms (Gomes et al., 2018).

Co-simulation is used in ship design, vessels movement, system design and more. It is described as an appropriate way to run multiple scenarios for the analysis of maritime systems (Laesche et al., 2013). Such scenarios include the evaluation of algorithms for route planning and optimization, the efficiency evaluation of shipping lanes, the valuation of traffic management systems, the evaluation of risks like grounding and collisions and more (Dibbern and Hahn, 2014). Also, for maritime systems, the use of co-simulation is preferred to full-system simulation, because the latter has two main challenges. The complexity of the various physical and engineering aspects of a vessel makes it difficult to effectively simulate its behavior. At the same time, the existing simulation tools are very specialized, mainly developed for research and optimization of subsystems and lack interconnection capabilities.

### 2.5. Existing ICT architectures and standards in the maritime

The VesselAI project leverages technological advances and outcomes from other EU-funded projects in the context of ICT systems and applications in the maritime sector. Specifically, the VesselAI platform's building blocks are based on existing knowledge from projects, which are reported in Table 1. Also, this table describes how VesselAI platform differs from the existing EU-funded projects.

**Table 1:** Existing projects/architectures.

**Project**	**Description**
Big Data Ocean^a^ (Lytra et al., 2017)	Big Data Ocean was a EU-funded project that delivers a marketplace for maritime data sources and enables big data scenarios. In particular, semantic and linked data characteristics increase the value and extracted patterns to monetize maritime information. VesselAI exploits Big Data Ocean marketplace, and semantic mechanisms to enforce decision-making in VesselAI pilot use cases. Big Data Ocean focuses on maritime data monetization through the instantiation of a marketplace. On the contrary VesselAI re-uses the marketplace functionalities to support data exchange between stakeholders to support the knowledge exchange through anonymized data and trained models exchange.
DataPorts^b^ (Belsa Pellicer et al., 2022)	DataPorts provides a Data Platform in which transportation and logistics companies around a seaport are able to manage data like any other company asset, in order to create the basis to offer cognitive services. DataPorts provides insights for VesselAI services, especially for vessel behavior and modeling related to port operations. VesselAI collaborates with DataPorts by exchanging know-how and tools for the AI and cognitive services developed as well as collaboration in dissemination activities and stakeholder engagement.
datACRON^c^ (Vouros, 2017)	datACRON advances the management and integrated exploitation of voluminous and heterogeneous archival data and streaming data sources, so as to significantly advance the capacities of systems to promote safety and effectiveness of critical operations for large numbers of moving entities in large geographical areas, including maritime domains. VesselAI broadens the application spectrum of datACRON, targeting to a much wider variety of application. Moreover, VesselAI puts more focus on utilizing a combination of technologies (HPC, IoT, and big data), in order to open new high-fidelity vessel models for the proliferation of innovative application and business horizons in the maritime sector.
INFORE^d^ (Vodas et al., 2021)	INFORE's mission is to address the challenges posed by huge data sets and pave the way for real-time, interactive extreme-scale analytics and forecasting, focusing on three diverse application areas: maritime surveillance, financial forecasting and life science. The ability to forecast, as early as possible, a good approximation to the outcome of a time-consuming and resource demanding computational task allows quickly identification of undesired outcomes and save valuable amount of time, effort and computational resources. VesselAI re-uses and extends the developed techniques for trajectory data summarization and highly efficient vessel event detection.

^a^https://www.bigdataocean.eu/.

^b^http://www.dataports-project.eu/.

^c^http://datacron-project.eu/; https://cordis.europa.eu/project/id/687591.

^d^https://www.infore-project.eu/.

## 3. VesselAI concept and architecture

### 3.1. The VesselAI concept

**Figure 1:**
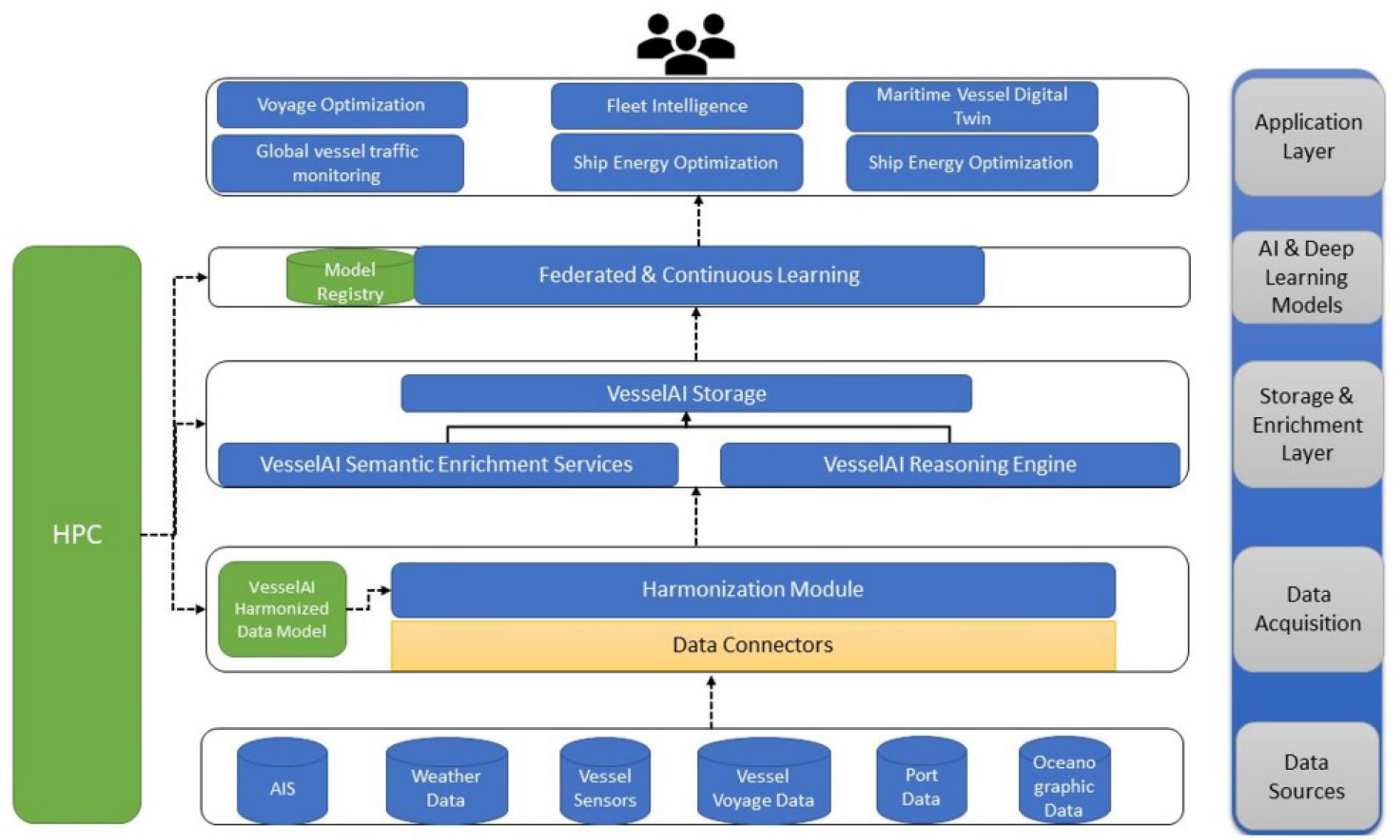
The VesselAI high level solution.

VesselAI leverages and combines existing frameworks and solutions in the areas of HPC, AI and big data to develop analytical services and decision-making procedures to support maritime digital twins and applications (Mouzakitis et al., 2022). The VesselAI components capitalize the amount of data that are generated from the maritime vessels (such as vessel sensor data, autonomous vessels data, AIS data) and combine them with data originating from weather & meteorological databases, satellite images, oceanographic data, and port data to support the machine and deep learning models training (Mouzakitis et al., 2023). The data management and data fusion mechanisms of VesselAI (Herodotou et al., 2020) support the inference functionalities in a wide range of use cases under different perspectives like the vessel traffic management (van Westrenen, 2014), vessel manoeuvering (Gil et al., 2020), fuel consumption optimization (Yan et al., 2021), collision avoidance (Mizythras et al., 2021) and other plethora of scenarios that will be extended under front-end applications utilized by maritime stakeholders. In general, VesselAI is an AI-oriented solution that tackles data processing and computational problems by leveraging state-of-the art HPC technologies, real time analytics and cloud technologies for creating data driven digital twins that offer (a) ship modeling for global vessel traffic monitoring and management, (b) optimal design of ship energy systems, (c) autonomous ships management in sea transport, and (d) weather routing and fleet intelligence. Figure 1 depicts the VesselAI concept.

The proposed VesselAI solution, relies on data stream workflows, AI and HPC acceleration technologies (Pyzer-Knapp et al., 2022) and on federated machine/deep learning algorithms (LSTM, RNN, ANN) to detect anomalies at global scale and monitor vessels and their direction at global scale (Han and Yang, 2021). Furthermore, model-driven and data-driven simulation scenarios are conducted, to design optimal ship energy systems (Trivyza et al., 2019). The simulation mainly focuses on the vessels operating conditions performing inferences by executing high performance analytical queries and data fusion procedures on a large number of energy-related features and subsystems to define the optimal conditions and practical operations (Niese et al., 2015) throughout a vessel's lifecycle. The VesselAI services leverage pretrained forecasting algorithms and vessel historical navigational data to predict anomalies compared to the normal traffic pattern and predict the next ship maneuvers. Finally, the VesselAI Analytical Services tackle the weather forecast uncertainty by enabling fleet intelligence services and functionalities for route updating and voyage optimization (Yu et al., 2021) to avoid excessing fuel consumption and to ensure overall ship safety. In this context the VesselAI deep learning models reuse historical vessel voyage data, weather data and AIS data to perform accurate predictions that reduce maintenance and fuel costs of operating vessels.

### 3.2. The VesselAI architecture

**Figure 2:**
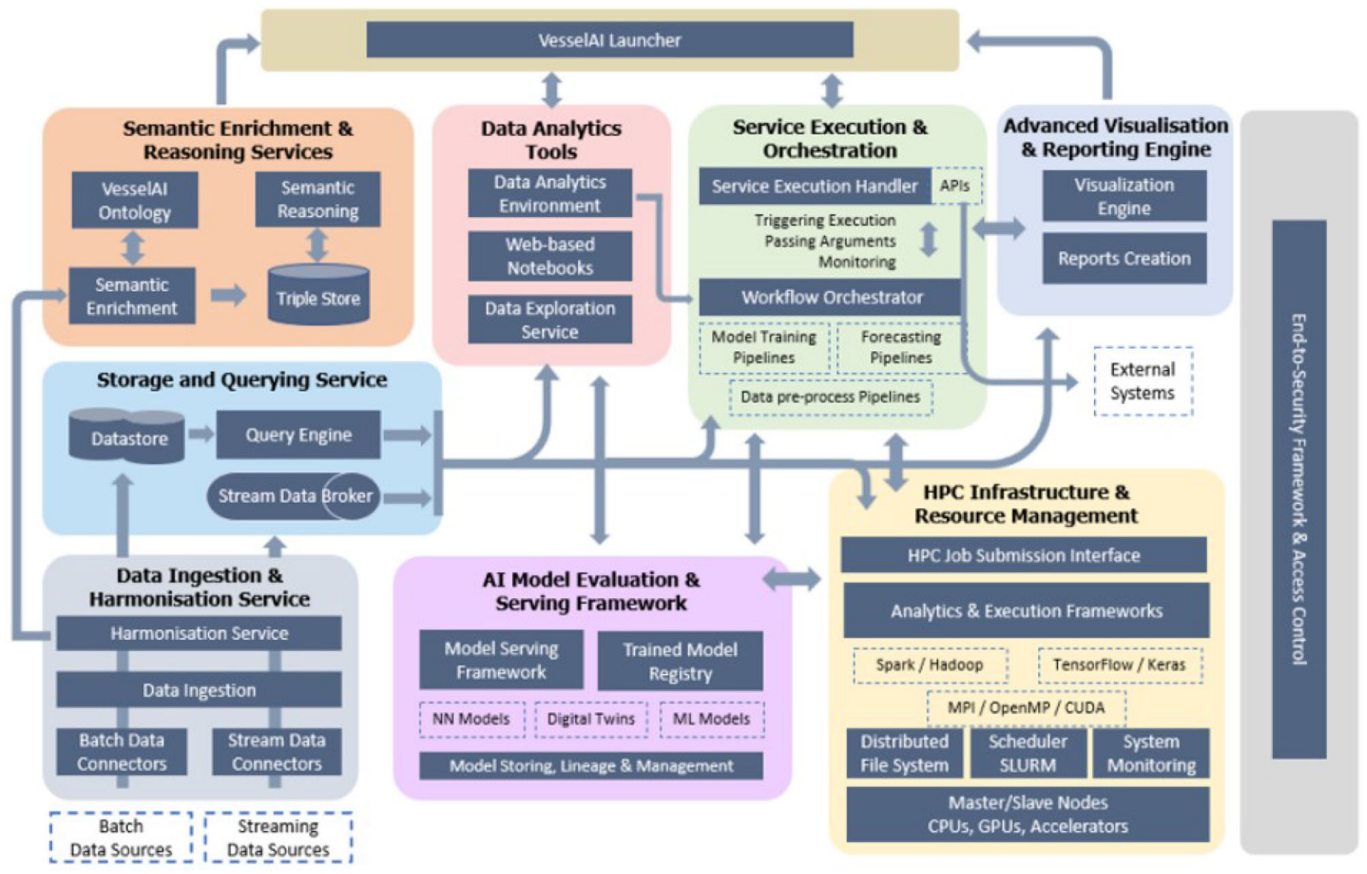
The VesselAI architecture.

Figure 2 demonstrates the VesselAI architecture. The depicted schema is an elaborated view of Figure 1 being the result of the technical specification elicitation process of the VesselAI platform. The VesselAI platform is consisted of the Data Services layer, the HPC Infrastructure and Resource Management layer, the VesselAI Model Serving layer, the Analytics & Visualization Services layer and finally, the VesselAI Launcher and Security Framework. In the following sections, each one of these layers will be analyzed and described.

#### 3.2.1. VesselAI data services

The VesselAI Data Services are responsible for the efficient ingestion, cleansing, harmonization, and enrichment of extreme-scale datasets stemming from different sources and targeting meaningful preprocessing tasks for machine and deep learning algorithm training. The VesselAI Data Services building blocks are the following:

##### 3.2.1.1. Data ingestion and harmonization service

The Data Ingestion and Harmonization Service is the entry point of data into the VesselAI platform. It ingests both batch and streaming data sources using different techniques suited to each data type (Paladin et al., 2022). Batch data sources can consist of local files, web resources or legacy databases, while streaming data sources consist of web streams and message brokers. Batch data can be preprocessed by the harmonization component before being loaded into the database or it can be directly ingested into the storage system. Stream data is immediately stored in the message queuing component and stored back into the message queue, providing a clean and standardized stream to the consumers. The use of a streaming message queue abstracts some of the challenges of streaming data while the use of the harmonization component provides cleaned and standardized data to be stored or processed by other services (Cazzanti et al., 2015).

##### 3.2.1.2. Semantic enrichment and reasoning services

The semantic enrichment component of VesselAI is mainly responsible for data transformation to common representation with clear semantics and data interlinking with data that originates from different data sources (Brüggemann et al., 2016). The process of data transformation generates data in RDF, which is the standard for semantics and linked data. In addition, the schema of the generated RDF data is defined by the VesselAI ontology, which provides a common vocabulary and representation of the knowledge domain (Santipantakis et al., 2022).

##### 3.2.1.3. Storage and querying service

**Figure 3:**
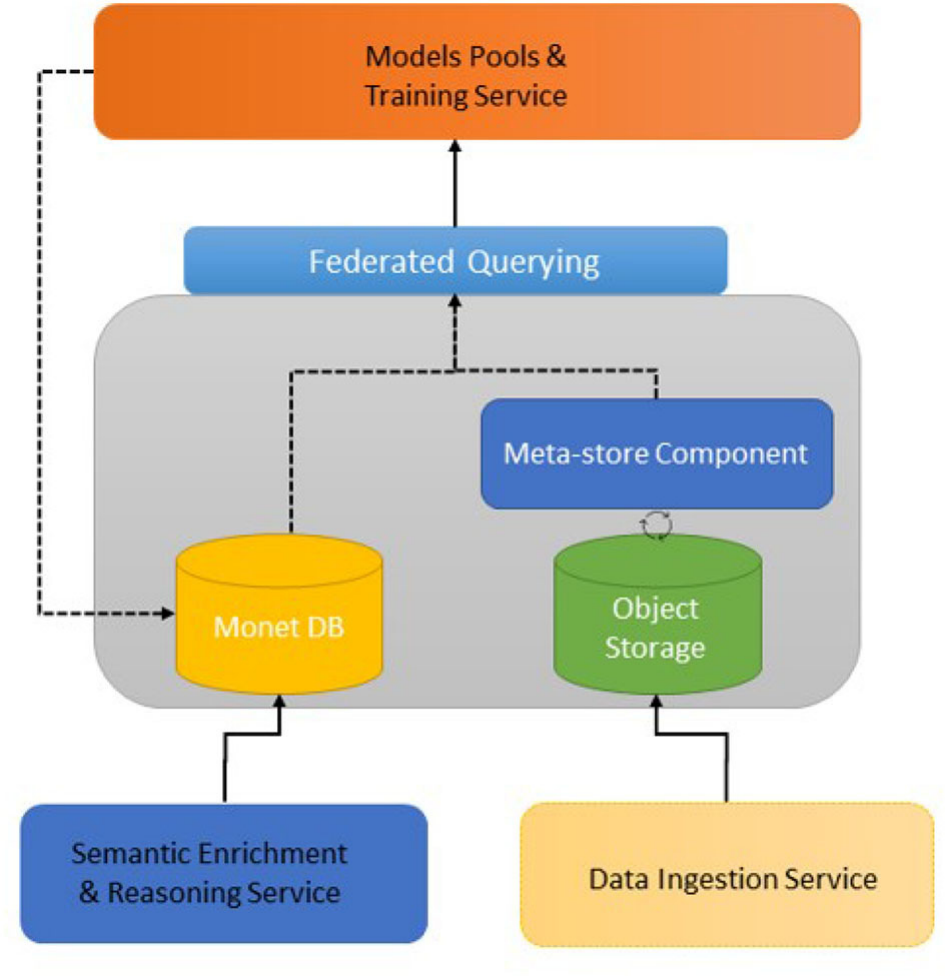
VesselAI storage and querying service.

The VesselAI Enriched Warehouse consists of an object storage where the data from the Harmonization Service and from the Semantic Enrichment Service are stored. In addition, a schema meta-store mechanism [Apache Druid (Correia et al., 2019)] is deployed on top of the object storage to query the persisted harmonized information. A relational database [MonetDB (Martinez-Rubi et al., 2015)] is deployed to store the prepared data for ML and DL model training. Finally, a Presto (Sethi et al., 2019) query engine is installed on top of Apache Druid (meta-store component) and MonetDB to support federated querying from both types of storages. The following image (Figure 3) depicts the architecture of the VesselAI Storage and Querying Service.

#### 3.2.2. VesselAI HPC infrastructure and resource management

High-performance computing (HPC) is an integral technology as it provides the much-needed computational power that big data-oriented technologies and applications require (Rajovic et al., 2016). The generic architecture of an HPC is built around a set of high-speed networks that interconnect computing nodes with storage devices as well as service nodes. The variation dimensions relate to the nature of the CPUs and associated computing accelerators in conjunction with the memory size at the level of the computing nodes as well as the distributed storage capacity and access methods at the level of the storage nodes. Interconnection networks vary according to their topology, routing strategy and are characterized by speed and latency. A preliminary study of the VesselAI use cases suggested a simple architecture made up of a limited number of fully connected CPUs which are associated with hardware accelerators through the standard PCIe protocol. To serve a wide spectrum of applications with varying memory requirements the compute node memory will adopt the standard formula based on the HPL/HPCG benchmarks, i.e., 2 GB (resp. 1GB)/ × 86 (resp. PowerPC) core. The overall storage capacity is estimated at about 300 TB according to the preliminary analysis of use cases requirements. An important point to mention here will be the presence of burst buffers (Herbein et al., 2016) to accelerate the access to storage devices. These storage capacities can be increased if required in the future.

As we are aiming for a relatively small configuration, separate interconnection networks dedicated to computation, storage and management nodes will be considered. InfiniBand interconnection network and high-speed Ethernet network will (Chen et al., 2019) be the preferred protocols for those interconnection fabrics. In association with the hardware architecture described above, a software stack is put in place to accommodate the implementation of applications in the system. The starting point is the SuperComputer SCS5 software stack [including Luster File System, SLURM Scheduler (Yoo et al., 2003), System Monitoring tools, Programming models, Libraries and debugging tools] developed by Bull as the basic operating layers of its HPC systems. Upon this basic software stack, successive software layers dedicated respectively to specific acceleration technologies (e.g., FPGA/GPU compiler, driver, debugger), intermediate interface (e.g., Intel/OpenVINO, Intel/OneAPI dedicated to Intel Acceleration, etc.) and AI frameworks (TensorFlow/Keras) (Nguyen et al., 2019) will be added to efficiently support a variety of AI-based applications.

#### 3.2.3. VesselAI AI model serving, analytics and visualization services

This layer comprises the intelligence of the VesselAI ecosystem. The trained ML and DL models are stored in the VesselAI Model Registry and served to the application layer via the VesselAI Serving Framework. The Service Execution and Orchestration component is responsible for launching and submitting model training jobs to the HPC Infrastructure and finally the Advanced Visualization & Reporting Engine is responsible for data exploration and training metadata visualization.

*TheVesselAI machine and deep learning models* for maritime applications and digital twins employ state of the art AI methods and techniques. Specifically, the artificial neural networks approach is used in forecasting vessel routes and traffic flows (Groba et al., 2018), detecting anomalies and vessel collisions. The AI models that are being implemented in the VesselAI framework concern route forecasting, traffic flow forecasts, collision detection, fuel consumption and others.

*The AI model registry and serving framework* is the sub-module responsible for hosting the trained VesselAI machine & deep learning models. In addition to the models themselves, the VesselAI Model Registry (Zaharia et al., 2018) stores metadata about the data and the training jobs used to create the model. The base technology that is used as a model registry is the MlFlow open-source component. The Serving Framework functionalities are also based on the MlFlow Registry that exposes the inference and prediction capabilities of the trained models as REST APIs.

*The service execution and Orchestration component* is responsible for managing the execution of analytical pipelines in the context of the VesselAI platform. The core component is the Service Execution Handler, which encapsulates the definition of multiple analytical services that are used to train AI and digital twin models (Anwar et al., 2020), and then use the trained models to perform inferences and generate forecasts and optimization outcomes. In addition, it orchestrates the whole lifecycle of the execution of the analytical pipelines (Hojaji et al., 2019), accepting the execution requests and input arguments, triggering the execution of the analysis, and finally serving the results by leveraging the VesselAI Model Registry & Serving Framework functionalities. The Service Execution Handler, as mentioned above, which is implemented on top of Apache Airflow, has integrated different data storages (MonetDB and MongoDB) during VesselAI Development cycles/releases. Pythonic Dynamic Acyclic Graphs (DAGs) are enabled to define periodical jobs in the VesselAI that implemenent the following tasks:

- Connection with maritime data sources.
- Execution of data pre-processing and curation routines.
- Periodical Models Training (e.g., when new data arrive on VesselAI data storage).
- Perform trained models evaluation based on test/validation datasets.
- Serving of trained artifacts to VesselAI Platform User Interfaces (UIs).

The second component of the service is Apache Airflow that acts as the Execution Orchestrator. It contains the code of the analytical tasks, forms pipelines and manages execution.

*The advanced visualization and reporting engine* is responsible for providing user interfaces with visualization and reporting capabilities. This component is based on known visualization technologies, such as Apache Superset (Michele et al., 2019), but it will be extended with the necessary visualization and reporting mechanisms and strategies according to the VesselAI stakeholders needs and goals. The main goal is to enable users to select one or more visualization techniques, setup their own custom dashboards and produce insightful visualizations for specific Maritime Domain scenarios.

### 3.3. VesselAI launcher and security framework

**Figure 4:**
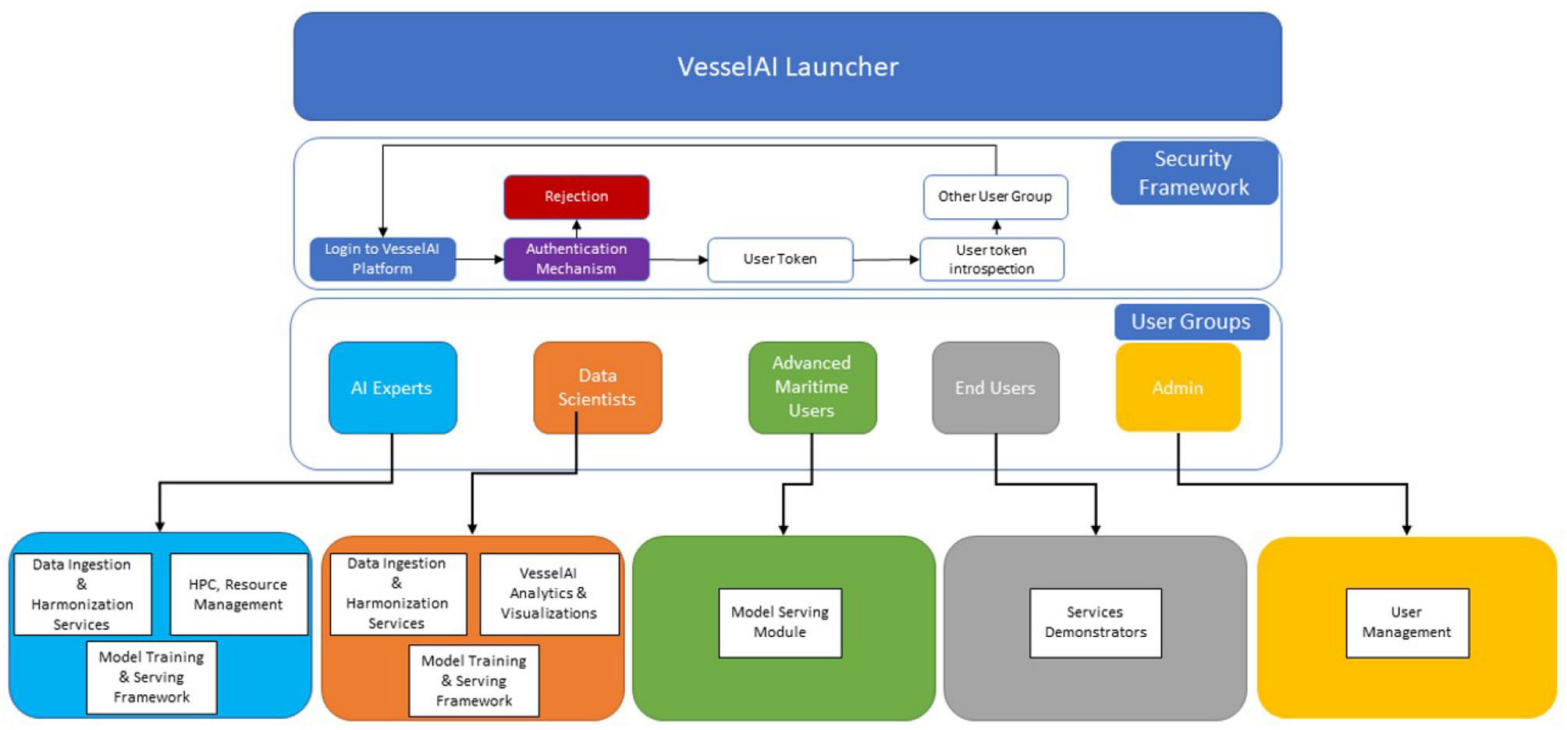
VesselAI launcher user groups and functionalities.

*The VesselAI Launcher* aims to enable efficient and cost-effective design and deployment of innovative services in the maritime industry, by leveraging state-of-the-art technologies in Data Engineering, AI and HPC (Yi and Loia, 2019). In essence, VesselAI's goal is to provide a novel holistic framework, capable of performing extreme-scale and distributed analytics for fuelling the next-generation of maritime digital twins and applications. Hence, the VesselAI solution envisions to provide a set of interoperable and modular data, AI, and HPC services, in an integrated package that has the potential to be deployed on the premises of each stakeholder (e.g., a maritime company) and operate independently, or interoperate with existing maritime applications of different architectures and deployment environments. The VesselAI platform will also include a visualization and data analytics environment that will offer a programming interface to a specific group of users, as well as access to dashboards/jupyter notebooks (Piazentin Ono et al., 2021) that will be created to serve as demonstration showcases for the deployed services. As such, the VesselAI Launcher was developed to “gather” all these services in the same environment. The VesselAI launcher will be a single page front-end application, through which the users will be able to sign in and obtain access to different services, based on the user group categorization. The VesselAI launcher will provide authentication and authorization functionalities for the users, as shown in Figure 4.

*The VesselAI security framework* is used to apply role-based access control over the VesselAI Launcher assets (services, data, frontend environments). Each VesselAI user group can apply specific actions over the Launcher UI. When users are logged into the platform, they get an OAuth2 Access Token (Sendor et al., 2014) which is then distributed to the Security Layer and introspected by its routines. The OAuth 2.0 protocol is among the most popular and widely used authorization/single sign-on (SSO) protocols and is also tailored for the new SSO standard, OpenID Connect (Fett et al., 2016). The Access Token Introspection procedure returns the user information, such as username, user roles and metadata. This information is sent back to the VesselAI platform and according to the user roles, it is decided if the logged-in user can access a specific VesselAI asset.

**Figure 5:**
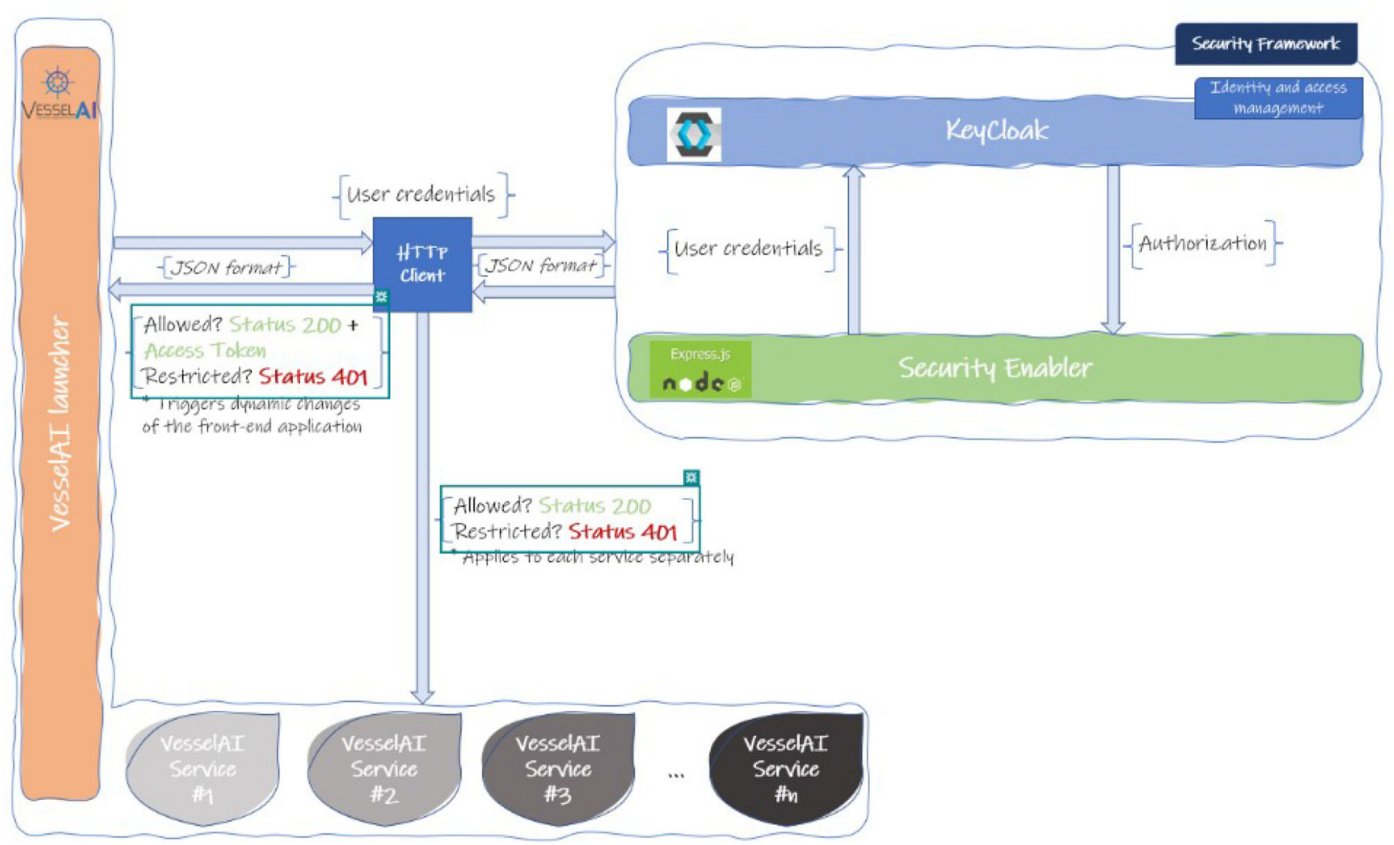
Security framework architecture.

The VesselAI Security framework/layer consists of two components as shown in Figure 5. KeyCloak, which is an open-source identity and access management framework is the first component, providing role-based access management with minimal development effort through implementation of the OAuth2 and UMA2 standards (Divyabharathi and Cholli, 2020). The second one is a custom component developed using Node.js, the Axios library, and express framework and acts as the Admin Middleware for KeyCloak. In fact, it is the orchestrator mechanism, which programmatically implements processes that the administrator of KeyCloak could potentially operate.

A workflow that depicts the scenario of a user must be established as the Homepage will not be accessible until the user is authenticated for security/consistency reasons.

The Oauth2.0/OIDC mechanism redirects a user from a web page to a KeyCloak server, where the user must provide his/her credentials (Sersemis et al., 2022). The credentials are transferred to KeyCloak, through the additional Security Layer (Security Enabler). KeyCloak, which is a user management platform offers the necessary mechanisms to decide if the credentials are valid. If they are, the user obtains an OAuth2 access token which, following the same logic, is passed, and stored to the user's browser, through the Security Enabler. On the contrary, if the credentials are not valid, an error message is sent, and the user is redirected to the login page.

After the login procedure, the user's access token which is stored in the browser's local storage is introspected by leveraging the Security Layer functionalities. After the introspection process the platform receives the user's information (roles, attributes, and other metadata) and by following a role-based access control policy it decides the VesselAI services and assets that the user can be given access to. In this way, the VesselAI Launcher will be dynamically altered to display links and relative resources according to the services that the user can have access to.

## 4. Discussion

To assess and evaluate the effectiveness of the VesselAI Architecture, there are some Key Performance Indicators (KPIs) which are described in detail in Table 2.

**Table 2:** KPIs for assessing the VesselAI architecture.

**KPI description**	**Target**
Time from decision to the implementation of a data processing framework handling large-scale and diverse data (streaming and batch).	70% decrease
Time from decision to the implementation of a ML and DL framework (preparation, training, serving).	70% decrease
Time from decision to the implementation of an HPC workflow for AI and big data workloads.	50% decrease
Time from decision for production to market launch for new maritime products and services based on vessel and vessel components models and behavior.	5% decrease
Increase in ML and DL predictions quality (accuracy, error rate, precision, recall and sensitivity).	10% increase

The attempt to combine the different technologies, i.e., distributed data storage and management, HPC Infrastructure and Resource Management, Machine and Deep Learning Models' training, and Machine Learning Operations, entails the following challenges:

- The data management procedures: Actions that include data curation, harmonization, semantic enrichment and storage of streaming data.
- HPC Infrastructure is the key ecosystem in VesselAI, where persisted data and database technologies must be hosted. Orchestration environments and modules, like Kubernetes, Cuda, Docker Swarm, and others, must enable functionalities that are computation and load intensive for optimizing data pre-processing and interoperability.
- The VesselAI Platform includes frameworks and components that offer scientific environments to VesselAI technical users (data scientits, data analysts), in the form of web-based notebooks, to train their machine learning and deep learning models. Artifacts after training phase are stored to VesselAI Models Repository, that acts as a storage of trained models. Machine Learning Operations (MLOps) are conducted on top of Models Repository to expose trained models via REST Services.

## 5. Conclusions and next steps

It is evident that the VesselAI Architecture presented under the scope of this paper provides a framework that enables state of the art techniques such as HPC, ML, and DL to facilitate maritime stakeholders in their everyday tasks, regardless of whether they possess advanced or non-advanced technical skills, and of course to create business value via the development and connection of the advanced maritime analytics services. The proposed architecture consists of multiple layers each one addressing the different requirements of the system. More specifically, the Data Services layer is responsible for the acquisition and enrichment of data originating from vessels, sensor, and/or noon reports. The Data Analytics layer is responsible for the analytics implementation, the model training and storage of the trained artifacts and finally the serving of the inference functionalities of the intelligence components. Finally, the VesselAI application layer is the central point of the VesselAI toolbox. The different stakeholders interact with the layers of the architecture to retrieve insights about their vessels regarding route forecasting, vessel route collision, creation and assembly of optimal energy systems and other functionalities that are exposed to the users through a library of AI models. AI acceleration technologies and HPC infrastructure are leveraged to provide insights and forecasts in real time.

Future steps of the current body of work will mainly focus on the deployment of the described architecture in several pilot platforms for testing its capabilities under different points of view and analyzing in greater detail the impact of the maritime digitalization process in selected scenarios and use cases.

## Data availability statement

The original contributions presented in the study are included in the article/supplementary material, further inquiries can be directed to the corresponding author.

## Author contributions

LI contributed to the writing of the manuscript on the conceptualization and to the design and implementation of the methodology. GT is responsible for the overall VesselAI architecture, the integration of all VesselAI components into the VesselAI platform, and a significant contribution to the manuscript for its revision. PK contributed to the conceptualization, the design and implementation of the methodology, the software development, and the writing of the manuscript. VM contributed to the design and implementation of the methodology and to the writing of the manuscript. GK was responsible for the design and implementation of the security layer of the architecture and offered a significant contribution to the manuscript for its revision. SM and DA contributed to the design of the research and supervised the findings of the work. All authors contributed to the article and approved the submitted version.
